# Comparison of Protein and Peptide Targeting for the Development of a CD169-Based Vaccination Strategy Against Melanoma

**DOI:** 10.3389/fimmu.2018.01997

**Published:** 2018-09-06

**Authors:** Dieke van Dinther, Henrike Veninga, Mirjam Revet, Leoni Hoogterp, Katarzyna Olesek, Joanna Grabowska, Ellen G. F. Borg, Hakan Kalay, Yvette van Kooyk, Joke M. M. den Haan

**Affiliations:** Amsterdam UMC, Department of Molecular Cell Biology and Immunology, Cancer Center Amsterdam, Amsterdam Infection and Immunity Institute, Vrije Universiteit Amsterdam, Amsterdam, Netherlands

**Keywords:** CD169, Siglec-1, sialoadhesin, tumor immunology, macrophage, T cell, antigen, cross-presentation

## Abstract

CD169^+^ macrophages are part of the innate immune system and capture pathogens that enter secondary lymphoid organs such as the spleen and the lymph nodes. Their strategic location in the marginal zone of the spleen and the subcapsular sinus in the lymph node enables them to capture antigens from the blood and the lymph respectively. Interestingly, these specific CD169^+^ macrophages do not destroy the antigens they obtain, but instead, transfer it to B cells and dendritic cells (DCs) which facilitates the induction of strong adaptive immune responses. This latter characteristic of the CD169^+^ macrophages can be exploited by specifically targeting tumor antigens to CD169^+^ macrophages for the induction of specific T cell immunity. In the current study we target protein and peptide antigen as antibody-antigen conjugates to CD169^+^ macrophages. We monitored the primary, memory, and recall T cell responses and evaluated the anti-tumor immune responses after immunization. In conclusion, both protein and peptide targeting to CD169 resulted in strong primary, memory, and recall T cell responses and protective immunity against melanoma, which indicates that both forms of antigen can be further explored as anti-cancer vaccination strategy.

## Introduction

Invasion of pathogens into the circulatory system can result in rapid development of disease. To prevent disease dissemination, the spleen, and lymph nodes sequester microbial, viral, and other nanoparticles from the blood or lymph fluid. In the marginal zone of the spleen and the subcapsular sinus of the lymph nodes, these entry sites are lined with phagocytic cells to trap invading pathogens (1, 2). One of the subsets of macrophages present at these locations are characterized by the high expression of CD169, also known as sialoadhesin or sialic acid-binding immunoglobulin-like lectin-1 (Siglec-1). CD169^+^ macrophages produce multiple cytokines and stimulate a variety of innate lymphocytes and together with these cells form a first line of defense after infection (3–7).

Next to their functions in innate immunity, CD169^+^ macrophages play a central role in the induction of both humoral and cellular adaptive immunity. Antigens captured by CD169^+^ macrophages are directly presented in intact form on their surface to follicular B cells and thereby stimulate germinal center B cell responses (8–13). We and others have observed antigen transfer from CD169^+^ macrophages to Batf3-dependent classical DCs (cDC1s) which in turn stimulate subsequent CD8^+^ T cell activation (14, 15). This antigen transfer between macrophages and DCs is facilitated by the CD169 molecule which functions as an adhesion molecule for sialylated molecules on DCs and binds strongly to cDC1s, (16). In addition, CD169^+^ macrophages appear to be able to directly stimulate CD8^+^ T cells after viral infection (15). As a result, antigen targeting to or infection of CD169^+^ macrophages stimulates strong CD8^+^ T cell responses that can lead to anti-tumor immunity (14).

Although the application of checkpoint inhibitors has dramatically improved the clinical outcome of several cancer types, most specifically melanoma (17, 18), still a significant portion of patients does not benefit from this form of immunotherapy. Since the response to checkpoint inhibitors is related to the presence of an anti-tumor T cell response, the induction of tumor-specific T cells by a vaccination approach could potentially increase the number of patients that will respond to the checkpoint inhibitor immunotherapy (18, 19). However, the optimal vaccination approach to induce CD8^+^ T cells is not yet established.

Because DCs are crucial for the activation of T cells, several previous experimental approaches focused on the *in vitro/ex vivo* generation of DCs that loaded with tumor antigens were to be utilized as a cellular vaccine. However, these cellular vaccines are very laborious and have not shown very strong clinical responses so far. *In vivo* targeting approaches are being developed in which antigens are directed to DCs through antibodies that bind to surface receptors specifically expressed on DCs. Several mouse studies have demonstrated the applicability of this approach for a number of surface receptors on DCs, most notably DEC205 and Clec9A/DNGR-1 (20–23), but (pre)clinical studies in humans are still necessary to determine which markers on (which) human DCs are most optimal for the activation of T cells. In our previous studies, we have shown that antigen targeting to CD169^+^ macrophages result in Ag presentation by DCs and the activation of strong CD8^+^ T cell responses in mice. In humans, CD169^+^ macrophages are also found in lymphoid organs and the numbers in tumor draining lymph nodes are positively related to longer survival in cancer patients. (24–28). Therefore, antigen targeting to CD169^+^ macrophages may form an attractive strategy to activate anti-tumor T cell responses in humans.

While a number of *in vivo* targeting studies used whole protein conjugated to antibodies, other studies utilized peptides containing only a CD8^+^ T cell epitope (21, 22, 29). Whole protein contains multiple epitopes to simultaneously induce CD4^+^ T cells, CD8^+^ T cell and B cell responses, while a peptide may only include single epitopes to induce CD8^+^ T cells and/or CD4^+^ T cells. Since helper CD4^+^ T and B cells enhance CD8^+^ T cell memory responses (30, 31), peptide targeting may lead to less than optimal long-term CD8^+^ T cells responses. However, next to these immunological differences, more practical considerations should also be taken into account. Some melanoma proteins are difficult to produce while a peptide has the advantage that it can easily be synthesized and will allow quicker implementation for future clinical applications. This especially may be advantageous when neoantigens will be used for vaccination. Because of these considerations, it should be determined if a peptide is sufficient to evoke a protective long-term anti-tumor immune response. We therefore compared whether CD169-targeting of whole protein compared to single peptide differed in the induction of specific T cell responses and subsequent tumor eradication. Our experiments show that peptide targeting is as efficient as protein targeting and could be implemented in a vaccination strategy for melanoma.

## Materials and methods

### Mice

C57Bl/6 mice were bred at the animal facility of the VU University Medical Center (Amsterdam, The Netherlands). Females between the age of 8–12 weeks were used for the experiments unless indicated otherwise. All mice were kept under specific pathogen-free conditions and used in accordance with local animal experimentation guidelines. This study was carried out in accordance with the recommendations of and approved by the “dierexperimentencommissie” or the “centrale commissie dierproeven.” Batf3 knockout mice were ordered form Jackson and bred in our facility.

### OVA and SIINFEKL conjugates

Ab-OVA conjugates were produced with SMCC-SATA mediated crosslinking as described previously (13, 14). In short, purified antibodies [αCD169 (MOMA-1), αDEC205 (NLDC-145), and a rat IgG2a isotype control (R7D4)] were functionalized with 5 equivalents of SMCC and endotoxin free OVA (Seikagaku) with 3 equivalents of SATA (N-succinimidyl S-acetylthioacetate, Thermo Fischer Scientific Breda) in phosphate buffer pH 8.5. Antibodies were desalted over PD-10 columns (GE Life Sciences Eindhoven) against phosphate buffer pH 7.2, and concentrated with centricon 30 (Merck Millipore Amsterdam) down to 300 μL. OVA-SATA was deprotected with 100 mM hydroxylamine hydrochloride (Thermo Fischer Scientific Breda) and desalted over PD-10 columns against phosphate buffer pH 7.2. After concentration of OVA-SATA with centricon 30 down to 200 μL, 6 equivalents OVA was added to antibodies while stirring. The antibody-OVA conjugates are incubated at room temperature for 1 h prior purification over sephadex 75 10/30 column.

Conjugation of SIINFEKL-eahx-lysine(biotin) peptide to antibodies was realized via a sulfhydryl based coupling. Briefly, antibodies were functionalized with 8 equivalents of SMCC [succinimidyl 4-(N-maleimidomethyl) cyclohexane-1-carboxylate, Thermo Fischer Scientific Breda] in phosphate buffer pH 8.5. After desalting over PD-10 columns (GE Life Sciences Eindhoven) against phosphate buffer pH 7.2 activated antibodies were concentrated with centricon 30 (Merck Millipore Amsterdam) down to 500 μL. 12 Equivalents of peptides in 50 μL DMSO was added to the antibodies and after 1 h incubation at room temperature conjugates were purified over sephadex 75 10/30 column (GE Life Sciences Eindhoven) according to manufacturer's HPLC settings.

### Immunization

Mice were immunized i.v. with 1 μg Ab:Ag conjugates in the presence of 25 μg purified αCD40 Ab (1C10) and 25 μg Poly(I:C). On the indicated days after immunization spleens and/ or blood were taken for processing. Boosts consisted of 1 μg free SIINFEKL peptide in the presence of adjuvants i.v. 28 days after primary immunization. 7 days after the boost spleens were taken for processing.

### Flow cytometric analysis

Single cell suspensions were stained in 0.5% BSA in PBS for surface markers after blocking Fc receptors with clone 2.4G2. For intracellular staining 0.5% saponine buffer was used. For macrophages and DC staining spleens were digested with 2WU/ml Liberase TL (Roche Diagnostics) in PBS in the presence of 4mg/ml Lidocaine hydrochloride monohydrate (Sigma) and 50 μg/ml DNAse (Roche Diagnostics) at 37°C. Samples were measured on the Cyan (Backman Coulter) or the Fortessa (BD) and analyzed using FlowJo software (Tree Star).

### Intracellular cytokine production

Splenocytes were incubated for 5 h with OVA_257−264_ in the presence of GolgiPlug (BD) for CD8 T cells and with OVA_262−276_ overnight with last 5 h in the presence of GolgiPlug (BD). Cells were fixed in 2% PFA and stained in saponine buffer for IL-2 and IFNy.

### Antibodies for flow cytometry and immunofluorescence

Antibodies specific for: CD-8a-488 (clone 53-6.7 Biolegend), IL-2-488 (clone JES6-5H4 eBioscience), CD169-488 (clones SER4 and MOMA-1 in house), CD11a-FITC (clone M17/4 eBioscience), CD11c (clone N418 eBioscience), CD44-FITC (clone HI44a ImmunoTools), CD11c-PE (clone N418 eBioscience), CD38-PE (clone 90 eBioscience), CD4-PE (clone GK1.5 eBioscience), CD8a-PE (clone 53-6.7 eBioscience), GL7-biotin (eBioscience), B220-ef450 (clone 6B2 eBioscience), KLRG1-ef450 (clone 2F1 eBioscience), CD127/IL7Rα-APC (clone A7R34 Biolegend), CD8a-APC (clone 53-6.7 eBioscience), IFNγ-APC (clone xM61.2 eBioscience), CD62L-PECy7 (clone MEL-14 Biolegend), CD8a-PECy7 (clone 53-6.7 eBioscience), CD4-PERCPCy5.5 (clone RM4-5 eBioscience), CD8a-PERCPCy5.5 (clone 53-6.7 Biolegend). OVA-488 (Invitrogen). H-2Kb-SIINFEKL Tetramers (LUMC, Leiden). LIVE/DEAD® Fixable Near-IR Dead Cell Stain Kit (Invitrogen) was used according to manufacturers' protocol.

### Tumor experiments

200,000 B16OVA cells were injected s.c. in 100 μl PBS, 3, or 7 days later mice were treated i.v. with 1 μg Ab:Ag conjugates in the presence of 25 μg purified αCD40 Ab (1C10) and 25 μg Poly(I:C). Tumor outgrowth was monitored by measuring tumor size (length, height, width) 3 times per week using a caliper. Volume of the tumor was calculated using the formula for volume of an ellipsoid (4/3 ^*^ π ^*^ ($\frac{1}{2}$l)($\frac{1}{2}$h)($\frac{1}{2}$w)). Humane endpoints were chosen based on tumor size (max. 1,000 mm^3^) or general appearance of mice. Tumor cell injection, i.v. vaccination, and tumor measurements were performed blinded, mice were appointed randomly to groups or were distributed according to an equal distribution of different tumor sizes among groups before treatment.

## Results

### Efficient peptide Ag targeting to CD169^+^ macrophages *in vivo*

**Figure 1:**
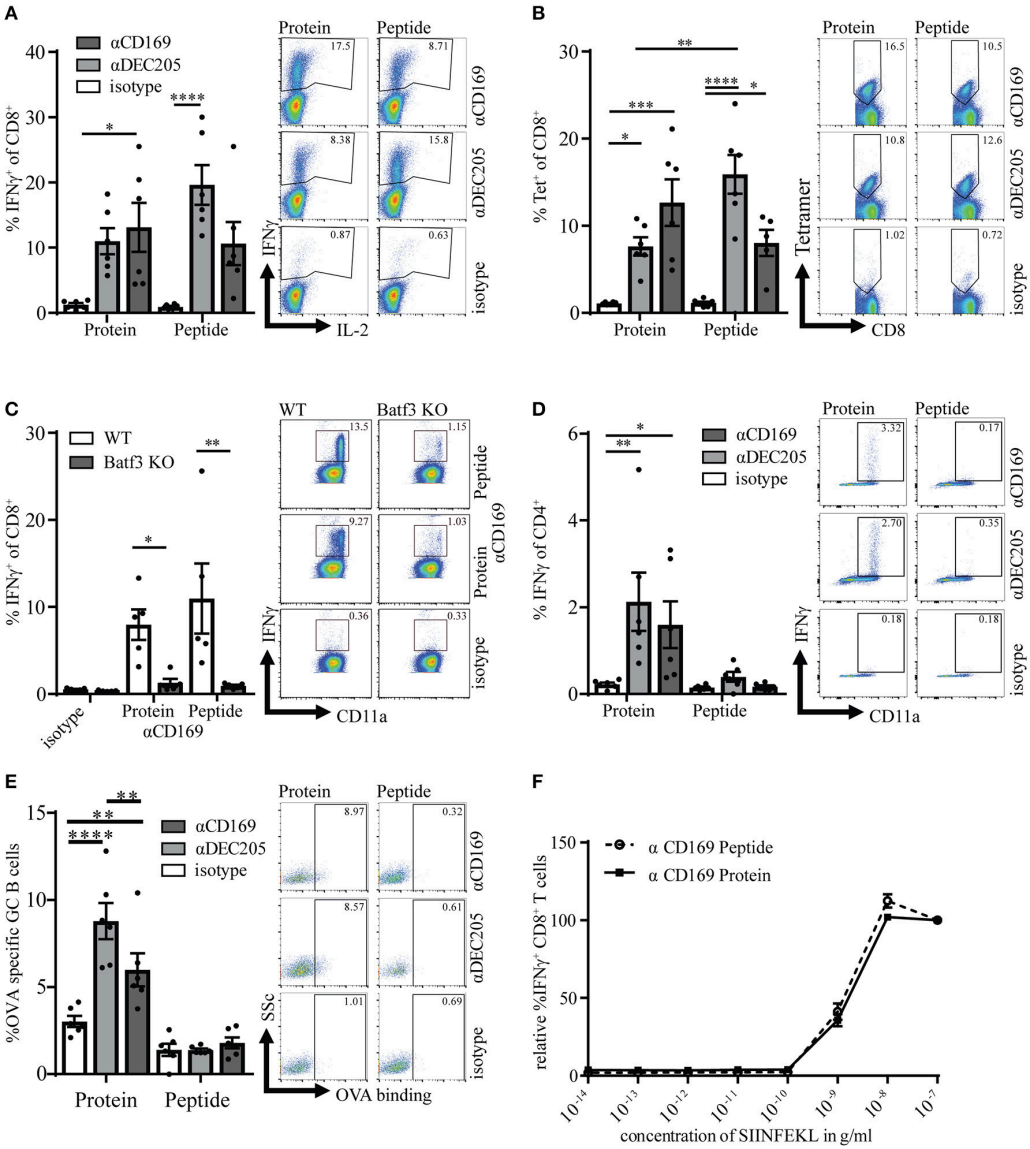
Targeting of protein and peptide to CD169 results in strong primary T cell responses. (**A**) Percentage of IFNγ producing CD8^+^ T cells after 5 h *in vitro* restimulation with SIINFEKL 7 days after immunization. (**B**) Percentage of CD8^+^ T cells binding H-2Kb-SIINFEKL tetramers. (**C**) same as in A in WT and Batf3 KO mice (**D**) Percentage of IFNγ producing CD4^+^ T cells after o.n. *in vitro* restimulation with I-Ab-restricted OVA_262−276_. (**E**) Percentage of OVA-binding germinal center (GL7^hi^CD38^−^) B cells (**F**) Relative percentage of IFNγ producing CD8^+^ T cells after 5 h *in vitro* restimulation with different concentrations of SIINFEKL. 100% is the IFNγ production after restimulation with the highest SIINFEKL concentration. (**A–F**) Splenocytes were taken 7 days after immunization with 1 μg Ab:Ag conjugates in the presence of 25 μg anti-CD40 Ab and 25 μg Poly(I:C). 1 representative experiment of 2–3 experiments is shown with 4–6 mice per group with one representative dotplot of each group. Statistical analysis one-way ANOVA with bonferroni's multiple comparison test *p < 0.05, **p < 0.01, ***p < 0.001, ****p < 0.0001.

In previous studies we have shown that targeting of ovalbumin (OVA) to CD169 on macrophages can result in T cell responses and that these T cell responses can reduce tumor outgrowth (14). Here, we compare the conjugation of the immunodominant CD8^+^ T cell epitope of OVA (SIINFEKL) to the whole protein, OVA, to verify whether a single peptide is sufficient to induce long-term CD8^+^ T cell responses and to inhibit tumor outgrowth. OVA and SIINFEKL were chemically coupled to specific Abs for targeting to CD169^+^ macrophages, to DEC205^+^ DCs as a positive control or to isotype control Abs (14, 29). The functionality and specificity of the CD169- and DEC205-specific Abs after conjugation was confirmed by fluorescence microscopy or by flow cytometry (Figures S1A–C). For the induction of immune responses *in vivo*, mice were immunized with 1 μg of the different Ab:Ag conjugates in the presence of 25 μg anti-CD40 Ab and 25 μg Poly(I:C). We observed a strong induction of Ag specific CD8^+^ T cells after both peptide and protein targeting to CD169^+^ macrophages and DEC205 (Figures 1A,B, gating strategy Figure S2A). The isotype control antibody induced low levels of OVA-specific CD8^+^ T cells as determined by intracellular IFNγ production or tetramer staining when compared to non-immunized naive mice (Figure S1D). The induction of specific CD8^+^ T cells was dose dependent (Figure S1E). As shown recently for OVA targeting to CD169 (16), also the CD8^+^ T cell responses induced by peptide targeting to CD169 relied on Batf3-dependent cross-presenting dendritic cells (DCs; Figure 1C). As expected only protein targeting induced OVA-specific CD4^+^ T cell and B cell responses (Figures 1D,E). The T cells activated after targeting either protein or peptide exhibited equal affinity for the SIINFEKL epitope as analyzed using an *in vitro* titration of the peptide (Figure 1F, Figure S2B). These experiments indicate that both peptide and protein targeting to CD169^+^ macrophages activate strong, cDC1-dependent, CD8^+^ T cell responses, while only the protein targeting induced OVA-specific helper CD4^+^ T cell and B cell responses.

### Targeting of OVA protein and peptide to CD169 results in efficient memory T cell induction

**Figure 2:**
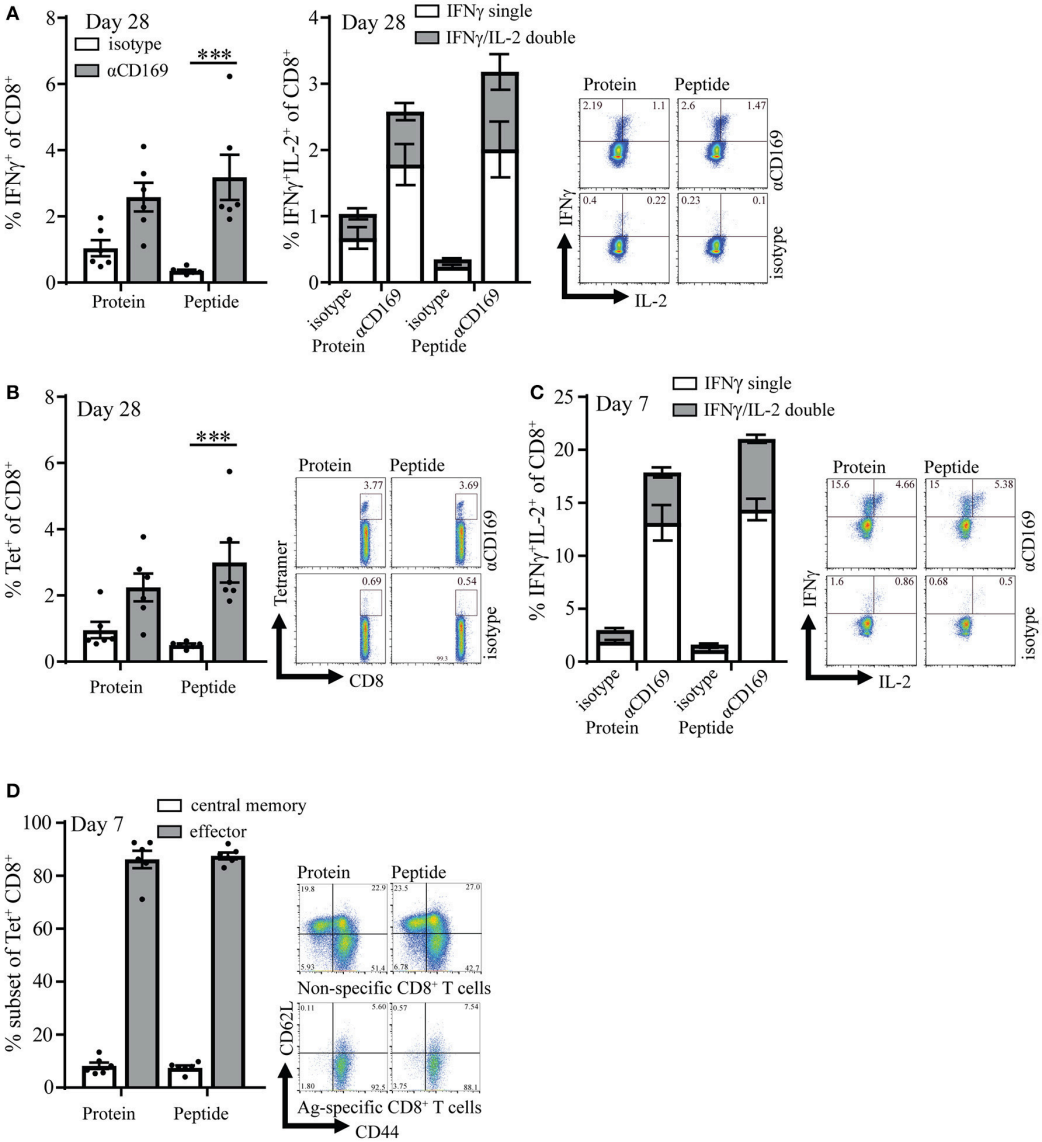
Targeting of protein and peptide to CD169 results in long-lasting T cell responses. **(A)** Percentage of IFNγ producing CD8^+^ T cells (left panel) and IFNγ single and IFNγ /IL-2 double producing CD8^+^ T cells (right panel) after 5 h *in vitro* restimulation with SIINFEKL 28 days after immunization. **(B)** Percentage of CD8^+^ T cells binding H-2Kb-SIINFEKL tetramers 28 days after immunization. **(C)** Percentage of IFNγ single and IFNγ /IL-2 double producing CD8^+^ T cells after 5 h *in vitro* restimulation with SIINFEKL 7 days after immunization. **(D)** Percentage of central memory (CD44^+^CD62L^+^) and effector (CD44^+^CD62L^−^) antigen-specific CD8^+^ T cells at day 7 after immunization and representative dotplots for Tet^+^ and Tet^−^ CD8^+^ T cells are depicted. **(A–D)** 1 representative experiment of 2 is shown with 6 mice per group with a representative dotplot for each group. Statistical analysis one-way ANOVA with bonferroni's multiple comparison test ***p < 0.001.

CD4^+^ T cell responses have been recognized as essential for the maintenance and recall capacity of memory CD8^+^ T cells (30, 32). To test if the absence of OVA-specific CD4^+^ T cell help during the immunization influenced the memory pool of specific T cells, memory CD8^+^ T cell responses were analyzed 28 days after immunization. At this time point the immune responses were still clearly measurable and we did not observe any difference between targeting of protein or peptide (Figures 2A,B, Figures S3A,B). Furthermore, similar percentages of CD8^+^ T cells could produce IL-2 on day 7 and day 28 (Figures 2A right panel, **C**) which is described to be indicative for memory T cells (33, 34). On day 7 most (~90%) of the specific T cells that were induced with this vaccination strategy showed an effector phenotype (CD44^+^ and CD62L^−^), while a small percentage (~10%) showed a central memory phenotype as shown by CD44 and CD62L coexpression (Figure 2D). Central memory phenotype CD8 T cells have been shown to have more proliferative capacity and better protective capacity than effector cells in infectious models (35). We did not observe differences in the generation of central memory CD8^+^ T cells when peptide and protein targeting was compared (Figure 2D).

**Figure 3:**
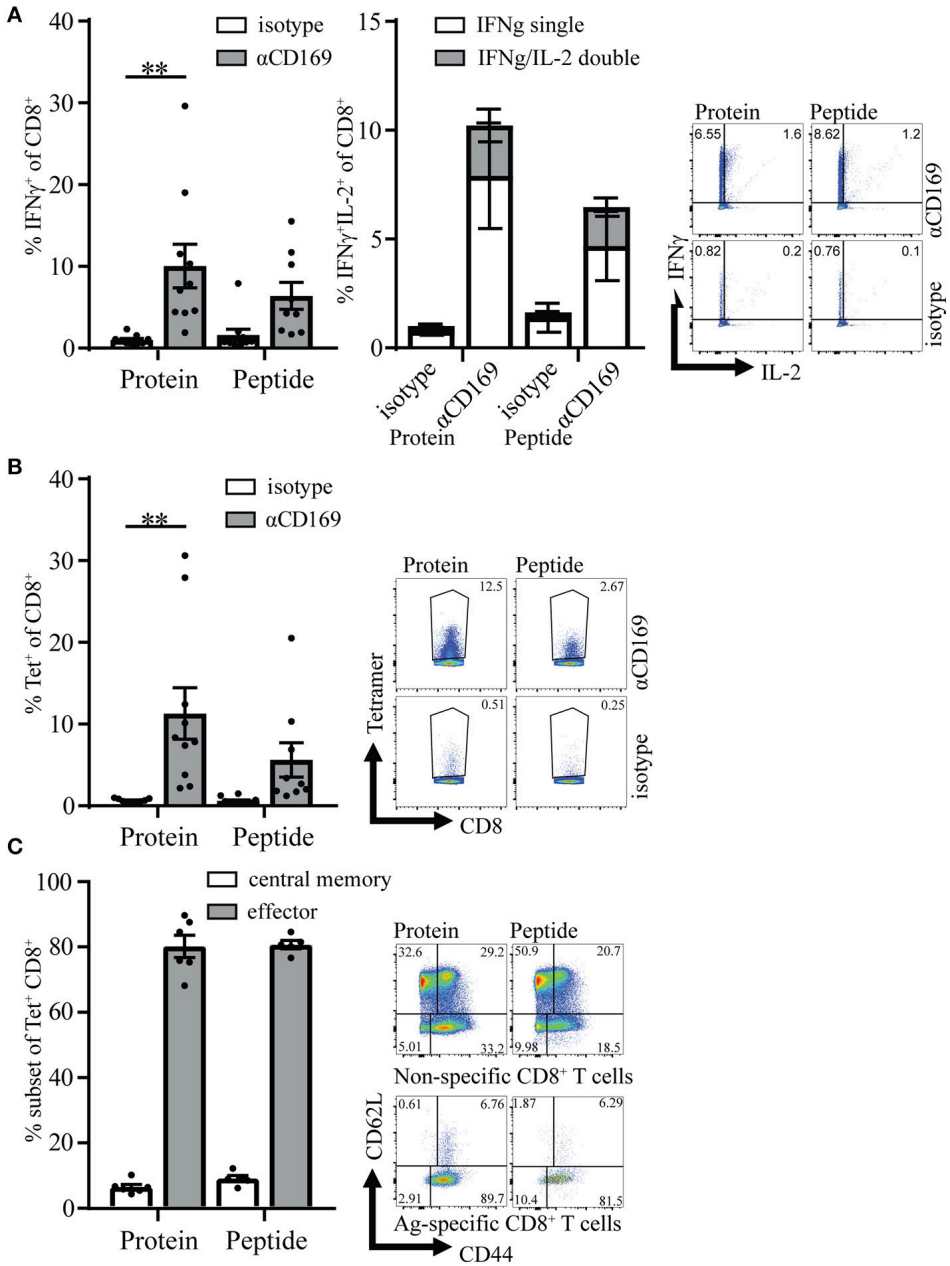
Memory CD8^+^ T cell responses after targeting antigen to CD169. Mice were initially immunized with indicated Ab:Ag conjugates and the immune response was boosted 28 days later with 1 μg free SIINFEKL peptide in the presence of adjuvants, 7 days after boost splenocytes were used for analysis. **(A)** Percentage of IFNγ producing CD8^+^ T cells (left panel) and of IFNγ single and IFNγ/IL-2 double producing CD8^+^ T cells (right panel) after 5 h *in vitro* restimulation with SIINFEKL peptide. **(B)** Percentage of CD8^+^ T cells binding H-2Kb-SIINFEKL tetramers 28 days after immunization. **(C)** Percentage of central memory (CD44^+^CD62L^+^) and effector (CD44^+^CD62L^−^) of tetramer^+^ CD8^+^ T cells and representative dotplots for Tet^+^ and Tet^−^ CD8^+^ T cells are depicted. **(A–C)** Combined results of 2 experiments is shown with 4–6 mice per group with a representative dotplot for each group. Statistical analysis one-way ANOVA with bonferroni's multiple comparison test **p < 0.01.

Four weeks after the primary response the mice were boosted with SIINFEKL peptide in the presence of adjuvant to determine the capacity to raise a secondary response. Primary immunization with peptide as well as protein led to memory T cell responses that generated strong recall responses as shown by intracellular IFNγ production and SIINFEKL-tetramer binding to CD8^+^ T cells (Figures 3A,B). The percentage of IFNγ/IL-2 double producing CD8^+^ T cells was similar for protein and peptide targeting and there was no difference in percentages of Ag specific T cells central memory and effector phenotype (Figures 3A right panel, **C**). These results indicate that peptide as well as protein targeting to CD169^+^ macrophages stimulate strong CD8^+^ T cell responses with equal potential for proliferation upon secondary encounter with antigen.

### Targeting of protein and peptide to CD169 results in efficient anti-tumor T cell responses

**Figure 4:**
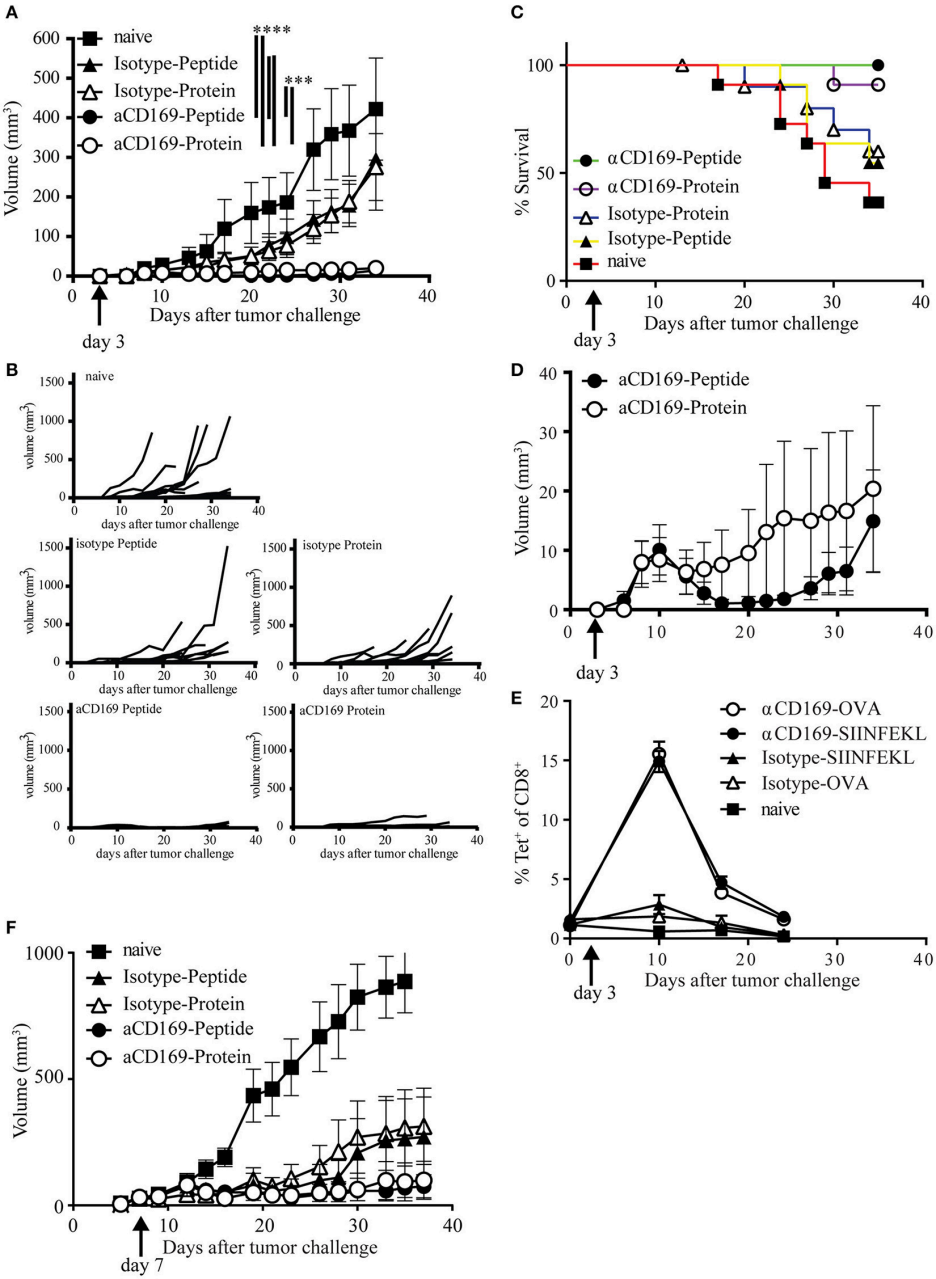
Targeting of protein and peptide to CD169 results in the induction of tumor reactive T cell responses. **(A–E)** Mice were inoculated with 200,000 B16OVA tumor cells at day 0, on day 3 mice were immunized with indicated Ab:Ag conjugates in the presence of anti-CD40 Ab and Poly(I:C). Tumor size was monitored three times a week and mice were sacrificed based on physical appearance or tumor size. **(A)** Tumor size on different days after tumor inoculation, mean ± SEM is shown. Two-way ANOVA with Bonferroni correction comparing all groups on the last day of the experiment, only significant differences are depicted. **(B)** Tumor size per group of each mouse after tumor inoculation **(C)** Percentage of surviving mice on indicated days after tumor inoculation. **(D)** Tumor volume as in **(A)**, but only showing the groups treated with the anti-CD169:Ag conjugates on a fitting scale. **(E)** Percentage of CD8^+^ T cells binding H-2Kb-SIINFEKL tetramers at indicated time points during the tumor experiment. **(A–E)** One experiment with 11 mice per group is shown. **(F)** Same as in **(A)**, with treatment on day 7 after tumor cell inoculation, when all mice had a visible tumor. Data of one experiment with 11–12 mice per group is shown. Statistical analysis one-way ANOVA with bonferroni's multiple comparison test ***p < 0.001, ****p < 0.0001.

To test if the CD8^+^ T cells induced by this vaccination strategy are able to kill tumor cells *in vivo*, we used a therapeutic vaccination setting. Mice were injected s.c. with melanoma B16OVA tumor cells and 3 days later the mice were immunized with OVA protein or SIINFEKL conjugated to isotype control Ab or antiCD169 Ab. Immunization with protein or peptide targeting to CD169^+^ macrophages was equally able to suppress outgrowth (Figures 4A,B). Since mice were sacrificed when the tumor size reached 1,000 mm^3^, prolonged survival was observed in those groups that received a vaccination (Figure 4C). The number of mice that had an established tumor (size > 2 mm^3^) during the course of the experiment was highest in non-treated and isotype-treated groups, while the number of mice that did not develop any tumor during the course of the experiment was highest in the protein targeted group. Interestingly, tumors initially grew in all the CD169-targeted groups the first 10 days (Figure 4D), but their growth was inhibited at day 10 which coincided with the peak in the CD8^+^ T cell response, as measured by the SIINFEKL-specific CD8^+^ T cells in the blood (Figure 4E). This was especially clear in the peptide targeted group (Figure 4D). To test if the vaccination strategy targeting protein and peptide to CD169 could result in T cell responses strong enough to suppress established tumors, mice with a visible tumor (average tumor size of 30 mm^3^) were treated 7 days after tumor inoculation. The progression of established tumors into a fast-growing tumor was suppressed after targeting protein or peptide to CD169 (Figure 4F, Figures S4A–C). Together these data show that the induction of SIINFEKL-specific CD8^+^ T cell responses by either protein or peptide vaccination results in efficient control of tumor outgrowth.

## Discussion

Vaccination approaches to induce anti-tumor CD8^+^ T cell responses should fulfill a number of requirements. First of all, sufficient numbers of effector CD8^+^ T cells should be activated to eradicate the existing primary tumor and/or metastases. Adoptive T cell transfer studies in both patients and mouse models have indicated that the number of transferred T cells is correlated with tumor regression (36, 37). Similarly, the number of T cells induced by vaccination approaches is predictive for their capacity to induce regression of existing tumors (38). Secondly, the differentiation stage of the activated CD8^+^ T cells is important. Central memory or memory stem cell T cells are better in eliminating tumors than terminal differentiated effector cells that are obtained by multiple rounds of restimulation (36, 39). Thirdly, vaccination approaches should induce T cells with the capacity to efficiently home to tumors, such as resident memory T cells (40, 41) and should not induce T cells that home back to the vaccination site (42, 43). Finally, vaccination should result in long-lived CD8^+^ T cell memory that will continuously eliminate outgrowth of tumor cells. Long term CD8^+^ T cell memory is critically dependent on CD4^+^ T cell help. Activation of CD8^+^ T cells in the absence of CD4 T cells can result in effector cells when sufficient inflammation is present, but these cells have defects in restimulation, do not generate secondary responses, and are called “helpless” T cells (44–46). Vaccination approaches using only MHC class I restricted tumor epitopes may have the risk of not generating long-term CD8^+^ T cell memory.

In our studies, we have evaluated the targeting of OVA protein and peptide antigen to CD169^+^ macrophages to induce anti-tumor CD8^+^ T cell responses. We observed very high frequencies of OVA-specific CD8^+^ T cells (more than 10% of total CD8^+^ T cells) after just one intravenous vaccination and targeting to CD169^+^ macrophages was as good as targeting to DEC205^+^ dendritic cells. This excellent induction of CD8^+^ T cell responses, could potentially be due to the fact that CD169^+^ very efficiently filter the blood and bind targeting antibodies. In addition, one of the unique characteristics of CD169^+^ macrophages is the capacity to preserve intact antigen on their surface for days, which enables presentation and transfer to B cells and DCs (11, 13, 14, 16). Together these characteristics may enhance the amount of antigen presented during a longer period of time than that obtained during direct targeting to DCs. The activation of high numbers of CD8^+^ T cells in just one immunization is also beneficial for their differentiation status as multiple rounds of Ag encounter lead to terminally differentiated cells with decreased potential for tumor regression (36).

Although CD4^+^ T cells specific for OVA were not generated in the peptide vaccination and are considered essential for long-term CD8^+^ T cell memory, we did not observe differences in the percentage of IL-2 producing or central memory phenotype CD8^+^ T cells after priming and restimulation. Also similar memory CD8^+^ T cell responses were generated when peptide and protein targeting were compared. Apparently our vaccination did not lead to a defect in memory T cell generation when peptides were used for targeting which could potentially be explained by two factors. First of all, we utilized a very strong adjuvant that mimics CD4 T cell help (anti-CD40 plus poly I:C) which makes additional CD4 T cell help dispensable (47–49). Indeed CD8^+^ T cell responses are efficiently elicited in MHC class II-deficient mice with our vaccination approach, demonstrating the dispensable role for CD4^+^ T cells with this adjuvant (data not shown). Several clinical trials in which agonistic anti-CD40 Ab is tested for its effect in solid tumors such as melanoma, non-small cell lung cancer and pancreatic duct adenocarcinoma sometimes in combination with checkpoint inhibitors have been started. The combination of agonistic anti-CD40 Ab with a vaccination strategy has not yet been explored in clinical trials and may be considered for neoantigen peptide vaccination strategies.

Secondly, we used rat antibodies to target our antigens to the CD169^+^ macrophages. These rat antibodies are likely to be immunogenic in mice and could contain helper epitopes for CD4^+^ T cells as has been described for anti-Clec9A/DNGR-1 antibodies (50). Cloning of the CD169-specific antibodies and the generation of recombinant mouse antibodies would be necessary to exclude this additional immunogenicity. However, although potential rat IgG2a-specific CD4^+^ T cells could be induced in the vaccination procedure, these helper CD4^+^ T cells were not involved in the OVA-specific anti-tumor response.

Both peptide and protein targeting to CD169 stimulated potent anti-tumor CD8^+^ T cell responses that were as efficient to prevent outgrowth of B16-OVA melanoma cells, indicating that the generated OVA specific CD8^+^ T cell homed to the tumor. Interestingly, Batf3-dependent cDC1 have been shown to promote the generation of tissue resident memory T cells (40, 51). Batf3-dependent cDC1 also are known to facilitate CD4^+^ T cell help and efficient long-term memory CD8^+^ T cell generation (52, 53). Apparently, antigen presentation by cDC1 is crucial for optimal memory CD8^+^ T cell responses with the capacity to home into tissues. We previously showed that targeting protein antigens to CD169^+^ macrophages transferred antigen to cDC1 and cross-primed CD8^+^ T cells (14, 16). Here, we show that also peptide targeted to CD169^+^ macrophages required Batf3-dependent cDC1s to stimulate CD8^+^ T cell responses, which may explain the observed similar capacity of peptide and protein antigens to stimulate effector, memory, recall CD8^+^ T cell responses and the capacity to prevent tumor outgrowth.

A recent study has demonstrated that mouse tumors contain increased numbers of CD169^+^ antigen presenting cells with characteristics of both DCs and macrophages that are able to home to lymph nodes and to cross-present tumor cell-derived Ag (54). Since conventional DC numbers may be limiting in tumors, Ag targeting to CD169^+^ cells in tumor-bearing individuals may potentially have more impact than Ag targeting to conventional DCs. Although the structure of the human spleen is different from mouse spleen and lacks a marginal zone, CD169-expressing macrophages are found in perifollicular sheaths surrounding small capillaries, also located in close contact with B cells (28). These structures would also be optimally suited for capture of antigens from blood by macrophages and presentation to B cells and potentially also to DCs. Strategies that target to human CD169 molecules may therefore also be efficient in directing antigens to the right lymphoid structure for the activation of anti-tumor immune responses and should be further explored as a vaccination strategy in humans.

## Author contributions

DvD, HV, and JdH experimental design. Experiments were conducted by DvD, HV, MR, EB, LH, KO, and JG. HK synthesized conjugates. Data analysis by DvD, HV, and MR. The manuscript was written by JdH and DvD and edited by all authors.

### Conflict of interest statement

The authors declare that the research was conducted in the absence of any commercial or financial relationships that could be construed as a potential conflict of interest. The reviewer PVE and handling Editor declared their shared affiliation.
