# Supplementary figures and images for: Comparison of Protein and Peptide Targeting for the Development of a CD169-Based Vaccination Strategy Against Melanoma
Source: Front Immunol. 2018 Sep 6;9:1997. doi: 10.3389/fimmu.2018.01997 (PMC6135888; doi:10.3389/fimmu.2018.01997)

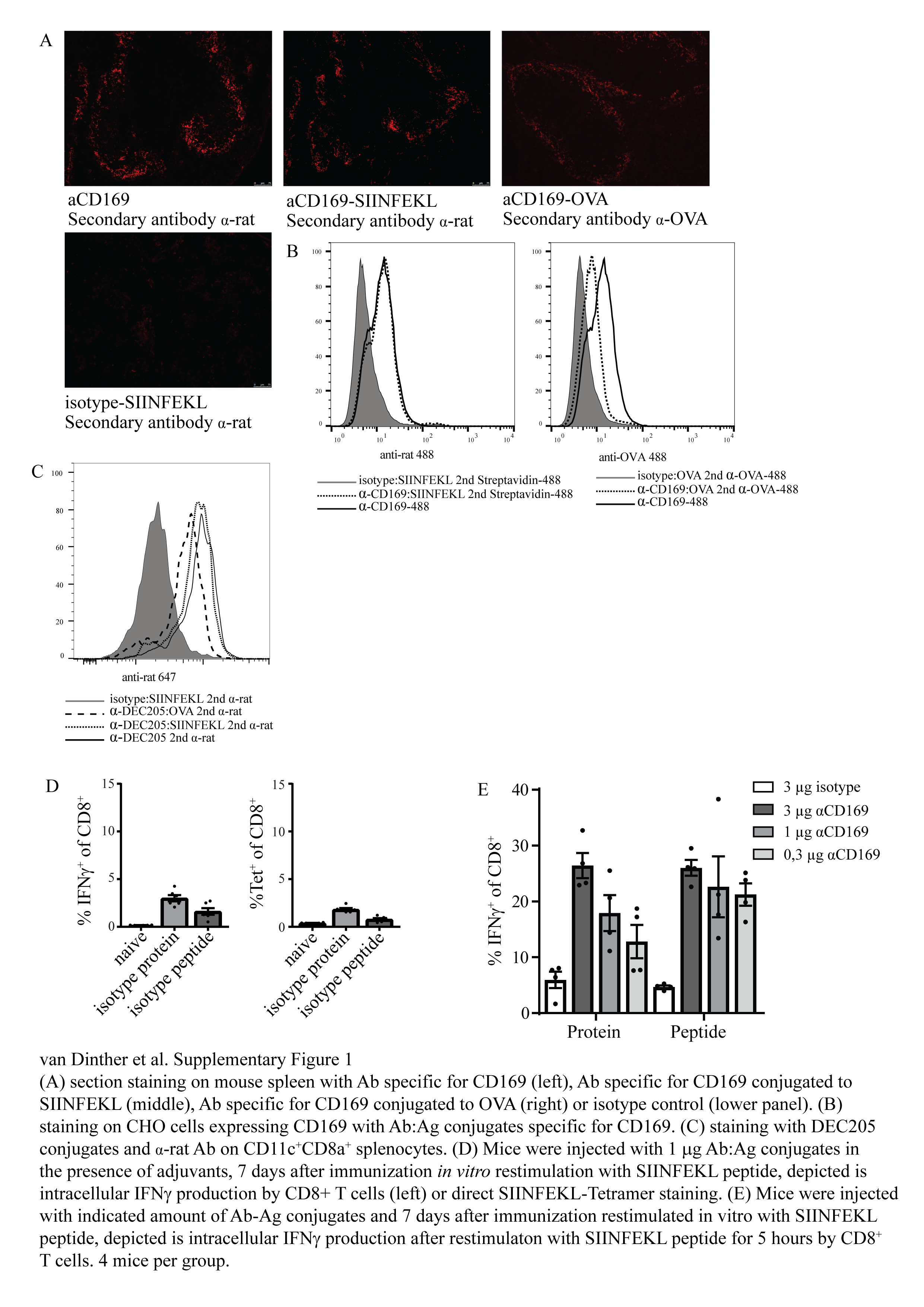

Supplement: Supplementary file 1 [file Image_1.TIF]

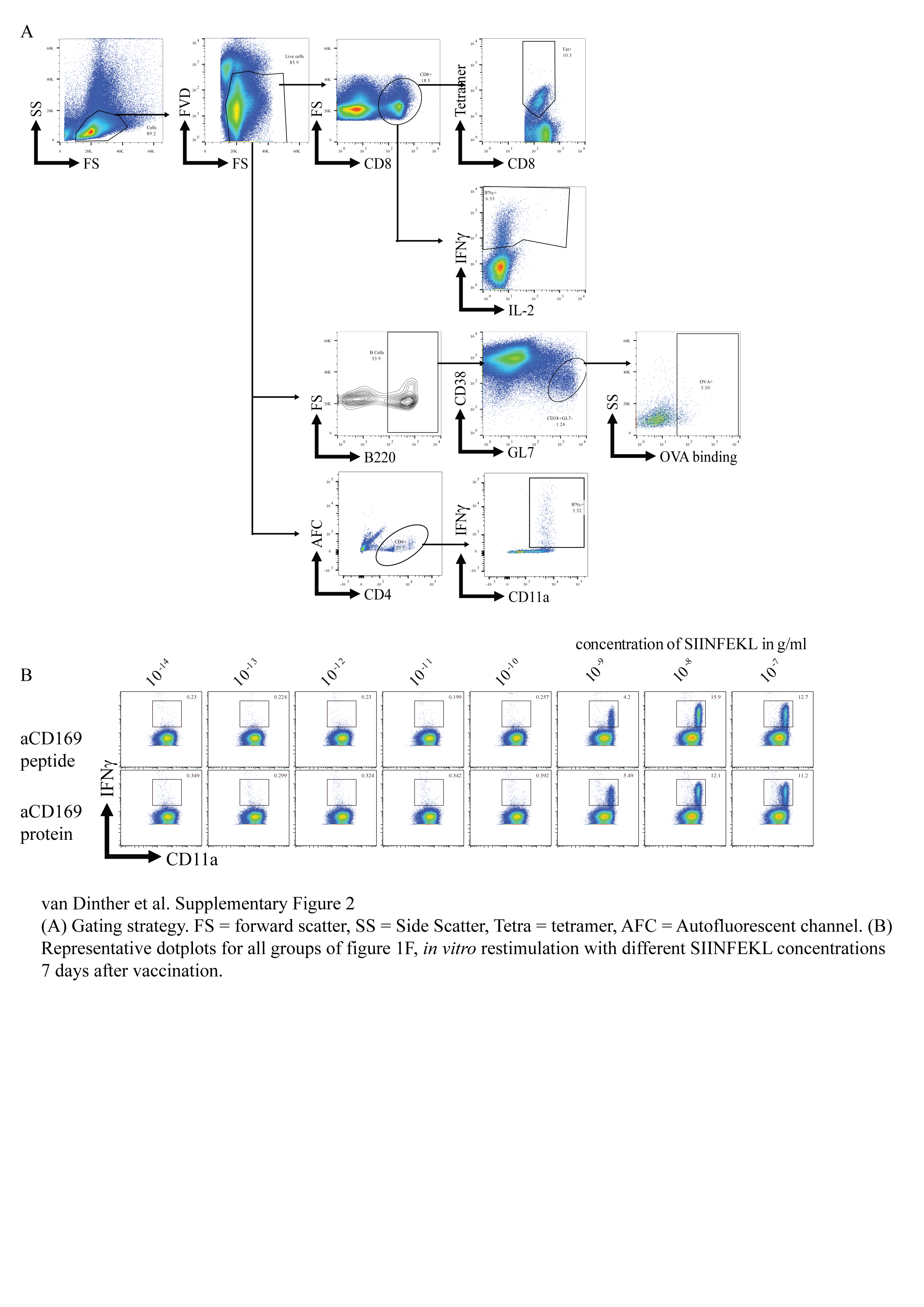

Supplement: Supplementary file 2 [file Image_2.TIF]

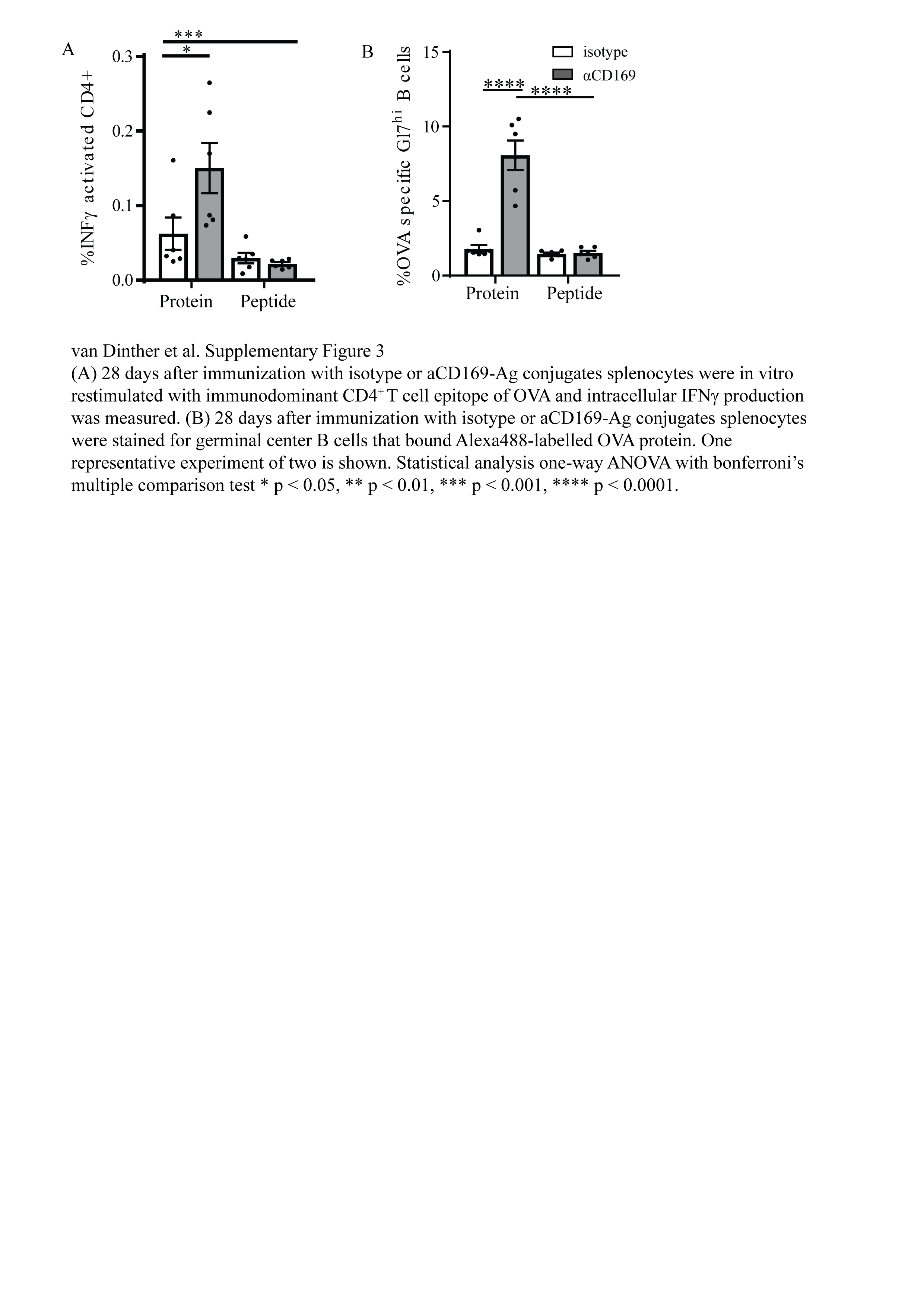

Supplement: Supplementary file 3 [file Image_3.TIF]

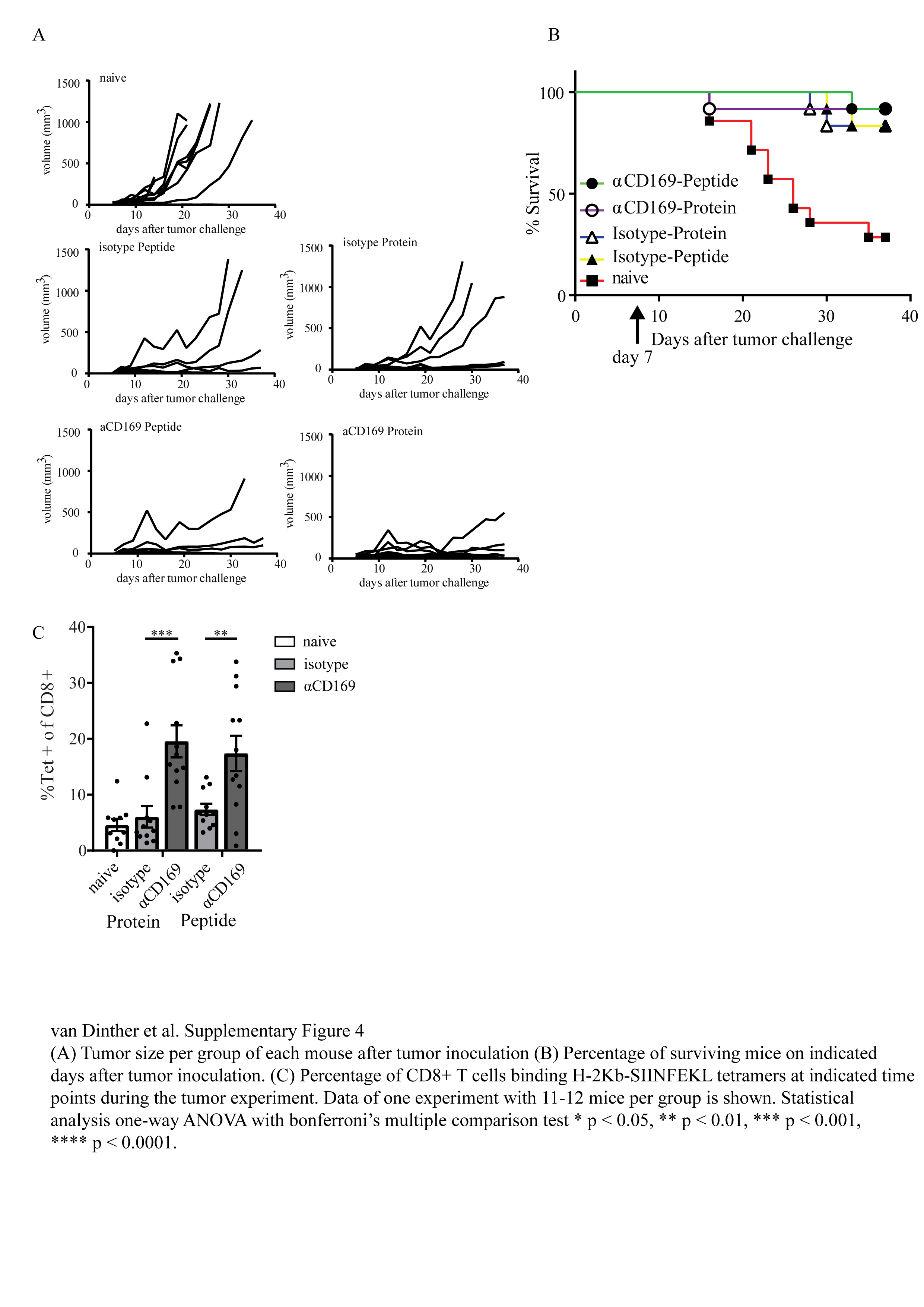

Supplement: Supplementary file 4 [file Image_4.TIF]
